# The Role of Study and Work in Cannabis Use and Dependence Trajectories among Young Adult Frequent Cannabis Users

**DOI:** 10.3389/fpsyt.2013.00085

**Published:** 2013-08-09

**Authors:** Nienke Liebregts, Peggy van der Pol, Margriet Van Laar, Ron de Graaf, Wim van den Brink, Dirk J. Korf

**Affiliations:** ^1^Law Faculty, Bonger Institute of Criminology, University of Amsterdam, Amsterdam, Netherlands; ^2^Trimbos Institute, Netherlands Institute of Mental Health and Addiction, Utrecht, Netherlands; ^3^Department of Psychiatry, Academic Medical Centre, University of Amsterdam, Amsterdam, Netherlands

**Keywords:** frequent cannabis use, cannabis dependence, young adults, qualitative research, life course approach, longitudinal study, education, employment

## Abstract

Life course theory considers events in study and work as potential turning points in deviance, including illicit drug use. This qualitative study explores the role of occupational life in cannabis use and dependence in young adults. Two and three years after the initial structured interview, 47 at baseline frequent cannabis users were interviewed in-depth about the dynamics underlying changes in their cannabis use and dependence. Overall, cannabis use and dependence declined, including interviewees who quit using cannabis completely, in particular with students, both during their study and after they got employed. Life course theory appeared to be a useful framework to explore how and why occupational life is related to cannabis use and dependence over time. Our study showed that life events in this realm are rather common in young adults and can have a strong impact on cannabis use. While sometimes changes in use are temporary, turning points can evolve from changes in educational and employment situations; an effect that seems to be related to the consequences of these changes in terms of amount of leisure time and agency (i.e., feelings of being in control).

## Introduction

Cannabis is among the most widely used illicit drugs worldwide, with between 125 and 203 million last-year users worldwide (1). In the US approximately five million persons use cannabis on a (almost) daily basis (2), and in the European Union an estimated three million individuals are (almost) daily cannabis users, most of whom are aged 15–34 years (3). Frequent (daily or nearly daily) cannabis use and particularly cannabis dependence are associated with various mental health problems and impaired functioning (4–8).

Associations between cannabis use, education, and employment have been extensively studied. Longitudinal research has shown that adolescent cannabis use is related to poor educational performance and early school dropout (9); degree attainment and university attendance (10); and reduced occupational expectations, attainment, and stability (11). A review on young adult substance use concluded that many risk and protective factors for adolescents remain for young adults, but, given the changing social contexts, factors such as college attendance and job attainment are specific for young adults (12). Regarding later life outcomes, adolescent cannabis use is related to lower income and higher unemployment in young adulthood (5). Adult past year cannabis users are more likely to quit their job to take another job, to be unemployed between jobs and to have lower levels of employment than non-past year users, including never users (13). French et al. (14) found that weekly or more frequent cannabis use was negatively related to employment, but less frequent use was not. In a longitudinal Norwegian study, cannabis users (use at least once in the past 12 months) reported lower levels of work commitment than less frequent users, regardless of individual characteristics (15). More generally, Arria et al. (11) showed that persistent drug users (at least once in every year studied) were more likely to be unemployed than non-users, and that part-time workers were more likely than full-timers to be drug dependent. Finally, Reed et al. (16) found that high job strains and low job control increased the risk on drug dependence. Together these findings suggest the presence of a reciprocal relationship between (changes in) occupational activities and (changes in) drug use and dependence, with changes in occupational activities leading to changes in drug use/dependence and changes in drug use leading to changes in occupational activities. However, little is known about the mechanisms responsible for these changes. One classical possible mechanism that could underlie this relationship is the “amotivational syndrome,” as it has been proposed that heavy cannabis use would cause (temporary) cognitive impairment including diminished motivation and memory, lack of interest, and concentration problems. However, these symptoms may as well be an outcome of other factors, such as depression, and no clear evidence until now supports this association (9, 17, 18).

Life course theory considers transitions such as changes in education and work as potential turning points in explaining desistance from deviance (19). Turning points are preceded by life events, which can be abrupt or gradual. Abrupt life events make sudden, sharp distinctions between past and future. Most events, however, are more gradual, and are part of a process. Life events could (objectively) be categorized as positive or negative, but their (subjective) meaning as positive or negative depends on how they are evaluated by the person experiencing them (19). Consequently, similar events can have different meanings for different individuals. When life events lead to a lasting change over time or a redirection of an individual’s course of life, including changes in deviance, they are considered turning points (20). Thus, turning points can only be identified in retrospect (21, 22). In life course theory, changes in deviance over the life course are explained within the context of age and maturation: most deviant behaviors peak in adolescence and young adulthood and then decline (19, 23). Employment has the potential to decrease deviance, because strong ties with work and informal social control could get an individual’s life (back) on track; not the job *per se*, but the commitment and stability associated with work can reduce deviance (19). Also, employment limits one’s time, thereby practically reducing opportunities for deviant activities (23).

Other researchers have emphasized the role of personal factors, such as “agency” in life events and desistance [cf. (24)]. In short, human agency refers to free will and (feelings of) control over one’s life, and contributes to how life events are experienced and might change into a turning point (20, 24). When using the concept of agency in this study, we follow Teruya and Hser (20), who defined it as “the amount of personal choice and control over decision making individuals feel they have,” and that “shapes their perceptions and the outcomes of life events and transitions and may contribute to the differential effects that the same life event may have on different people.” [(20) p. 4].

Although life course theory often concerns criminal careers and desistance from crime, we assume that it also applies to cannabis use careers, since largely similar processes are involved [cf. (25)]. Life events thus can become turning points when redirecting an individual’s path in substance use or dependence. In life course theory, employment, especially stable employment, is considered as one of the factors most commonly associated with desistance. The potential of employment to become a turning point is influenced by job characteristics and human agency (16, 20, 24).

Several of the earlier studies on drug use, education, and employment refer to any use in the last 12 months, which could range from only once to daily use. Consequently, it remains unclear to what extent frequent drug use, including cannabis use, is related to study and work. Probably more important is the need to better understand how and why frequent young adult cannabis users change their use, how these changes are related to transitions in and out of cannabis dependence, and how these changes and transitions are related to changes in study and occupational activities. Employment trajectories can have turning points with an impact on cannabis use and dependence, but cannabis use can also influence employment (26). To better understand the natural course of frequent cannabis use of young adults and the relation with education and work, our objectives in the current study are (1) to explore in-depth the meaning and role of education and work in using cannabis in general; (2) to analyze the relationship between events in these domains and changes in cannabis use; and (3) to analyze the role of occupational events in changes in cannabis dependence trajectories. We decided to use a qualitative approach, because the dynamics and the processes underlying the relationship of educational and work with cannabis use and dependence trajectories cannot be adequately addressed with quantitative methods and because personal narratives and in-depth interviews are deemed to improve our understanding of the processes and the context involved with these changes. This study is among the first to qualitatively capture the natural course and transitions in frequent cannabis use and dependence in young adults.

## Materials and Methods

### Study design

**Figure 1:**
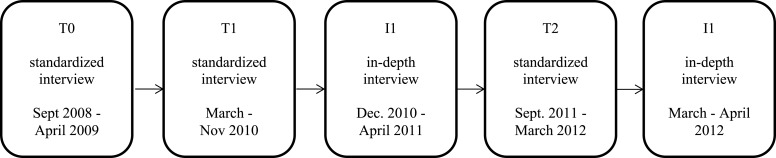
**Timeline of CanDep data collection**.

The current (qualitative) study is part of a broader longitudinal study (CanDep) on cannabis use and transitions in cannabis dependence in young adult frequent cannabis users [see for details (27)]. Figure 1 displays an overview of the different (quantitative and qualitative) interviews in the study. In brief, at baseline (T0, September 2008–April 2009) 600 frequent Dutch cannabis users (>3 days cannabis use per week in the past 12 months) aged 18–30 years were recruited in coffee shops and through respondent-driven sampling and interviewed [see for details (28)]. Participants were monitored for 3 years, with two follow-up interviews and six intermediate updates by e-mail or phone. At T0, DSM-IV diagnoses of 12-month cannabis dependence were assessed with the Composite International Diagnostic Interview (CIDI 3.0). After 18 months (T1, March–November 2010) and 36 months (T2, September 2011–March 2012) participants were interviewed again, including an assessment of their cannabis dependence status since the previous interview. At T1, four trajectories in cannabis dependence were distinguished: persistent non-dependent, persistent dependent, transition from dependent to non-dependent, and transition from non-dependent to dependent. At T2 the number of trajectories extended to eight.

In an additional qualitative sub-study, the dynamics underlying the changes in cannabis use and the transitions in cannabis dependence were investigated with special emphasis on study and occupational changes. We conducted life story interviews, in which users can express themselves through their narratives and thereby can improve our understanding of the processes and the context involved in these changes [cf. (24, 29, 30)].

From each of the four trajectories at T1, 12 participants were randomly selected, stratified for gender (8 male, 4 female), totaling 48 interviewees. At T2, these interviewees represented seven trajectories (Table 1). The first qualitative interview (I1) took place between December 2010 and April 2011, the second (I2) in March and April 2012. One participant could not be traced back at I2 and was excluded from the analysis, thus resulting in a final sample of 47 participants. While 47 participants is a small sample size for quantitative research methods, for qualitative methods this is not the case and a “small” sample size is considered more powerful in order to achieve depth [cf. (31, 32)].

**Table 1:** **Transitions in cannabis dependence status T0–T1–T2 and trajectory characteristics**.

Cannabis dependence trajectory	*n*	T0	T1	T2	Age T0 (mean)	Age first use (mean)	Cannabis career T0 (mean years)	(Near) daily use T0	(Near) daily use T2	Female
NNN	12	Non-dependent	Non-dependent	Non-dependent	21.8	14.4	7.4	6	3	4
NDN	7	Non-dependent	Dependent	Non-dependent	20.4	14.3	6.1	4	5	2
NDD	4	Non-dependent	Dependent	Dependent	20.5	14.2	6.2	3	2	2
DNN	10	Dependent	Non-dependent	Non-dependent	21.2	13.4	7.8	7	3	3
DND	2	Dependent	Non-dependent	Dependent	22.5	14.0	8.5	1	1	1
DDN	5	Dependent	Dependent	Non-dependent	19.8	13.4	6.4	3	2	1
DDD	7	Dependent	Dependent	Dependent	22.4	15.0	7.4	5	4	3
Total	47				21.3	14.1	7.1	29	20	16

### In-depth interviews

We conducted in-depth interviews, using a topic list that included questions about participants’ cannabis use career, i.e., changes in patterns of cannabis use, motives for change in cannabis use, and the occurrence of life events in various life domains. Interviewees were asked to recall changes in different life domains and in their cannabis use patterns between T0 and T1 and between T1 and T2, respectively, using detailed personal timelines [cf. (33, 34)]. One timeline referred to their cannabis use (including frequency and number of joints per occasion), the other timeline to life domains (including occupational life, i.e., education and employment). Both timelines were prepared before the interview and included data derived from the quantitative interviews and intermediate updates, which included questions about their cannabis use and occupational status (i.e., study and work). During the interviews these timelines were used as guidelines and elaborated in detail. Every interview started with an open question [“Thinking about your life between …. (T0 or T1) and … (T1 or T2), what has happened and what experiences have been important to you?”], and ended with a similar, but slightly different question [“Looking back at the period between … (T0 or T1) and … (T1 or T2), what experiences or processes do you consider to have had a (positive or negative) impact on your life and cannabis use?”]. While in the first in-depth interview (I1) participants’ entire cannabis career and life history until baseline (T0) were discussed, the focus in both in-depth interviews (I1 and I2) was on the period between the standardized interviews (T0–T1 and T1–T2 respectively). The study was approved by a Medical Ethics Committee. All participants provided written informed consent at the start of the study, acknowledging that their participation was voluntary. They all were assured that the interviews were confident and data was kept safe, separated from any personal information and that anonymity was guaranteed. Interviews took place at a quiet location; mostly at participants’ home and sometimes at the research institute. The interviews lasted between 1.5 and 3.5 h. After completion, participants received a financial compensation of €25.

### Analysis

All interviews were digitally recorded (with participant’s consent), transcribed verbatim, and imported into QSR Nvivo. Transcripts were analyzed combining deductive and inductive strategies. Codes and categories were partly developed beforehand, based on the literature [*a priori* coding: (35)]. In addition, new codes and categories evolved from the data, and new patterns emerged. Interview transcripts were read and reread to identify and link evolving codes, categories, and themes [pattern coding: (35)]. To guarantee anonymity, interviewees were identified with fictitious names and sometimes quotations were slightly adapted.

## Results

### Participants

Age of participants at baseline ranged from 18 to 30 years (Table 1). One third was female (by selection). Age at first use varied from 11 to 18 years (mean = 14 years).

At baseline, the length of cannabis use careers ranged from 1 to 15 years (mean = 7 years), for some with intervals of no use. At baseline (T0), 29 participants were (near) daily users (5–7 days per week) and the remaining 18 participants used on 3–4 days per week. During the study there was an overall decline in cannabis use frequency. At T2, 20 participants were (near)daily users, 19 participants used at least three times a week but not (near)daily, 3 participants had not used cannabis for 1 year or more and said they had quit permanently, and in the 5 remaining participants cannabis use varied from 1 day per week to less than monthly, including 3 participants who basically considered themselves as quitters, and had been using cannabis only a few times in the past year. Also quantity of cannabis used decreased, from on average 2.9 joints per using day at T0 to 2.4 at T2 (excluding three non-past year users). At T0, 24 participants were last-year cannabis dependent and 23 participants were non-dependent. At T2 this had changed to 13 dependent and 34 non-dependent participants. At baseline dependent and non-dependent interviewees were rather similar concerning mean age at initiation, mean age at baseline, gender and (near)daily use. At T2 cannabis dependent interviewees were more frequently (near)daily users than non-dependent participants, but also in NDN many participants were using (near)daily. Besides, relatively more females than males were dependent at T2.

### Education, employment, and commitment

Regarding occupational status, three categories were distinguished: students, employed and neither student nor employed. At baseline, almost two-thirds of the participants (31/47) were fulltime students. At the time of the last in-depth interview some had stopped studying without a qualification, some had graduated, but most (24) were still studying. Type of study varied from a vocational training to academic studies. Most students had a job on the side; some regularly 3 days a week, others every now and then when they felt in need of money. The most popular job among these students was working in cafes or restaurants. At baseline, about a quarter of the interviewees (11/47) were in paid fulltime employment (32 or more hours weekly). At T2 more than one third was employed (18/47), all but one fulltime. By then, some interviewees still worked at the same company, and sometimes had been promoted, while others had switched work several times during these 3 years. The growing number of employed participants is partly explained by participants graduating and then starting their job career, and partly by participants quitting their study unfinished and getting employed. The employment sectors were diverse, for example some worked in bars, others in academic professions. In the course of the study, one participant became unemployed at T2. At T0, the remaining five interviewees were neither student nor employed: three defined themselves as a fulltime parent, one was on social benefits, and one was in a reintegration program (with probation). Of these participants, the one on probation had become a student at T2 and the occupational status of the other four remained unchanged. To summarize, in the course of the study the number of students dropped from 31 to 24, the number of employed (almost exclusively fulltime) increased from 11 to 18, and the number of participants without study or work remained stable at 5.

Although the importance that student and employed interviewees attached to their study or work varied, only a few of them felt that it was not very important and that life was more about social activities and “having fun.”

*My study is somewhere on the background in my life. Of course it’s important for me to keep thinking about the future, and it plays a large role in that I have to go there a couple of times every week, and have to study for it, but if I fail a test, I fail a test, that doesn’t really bother me. (…) I’m not much of a scholar, for me the fun things in life are more important*. (Julius, I1, DNN)

however, most students attached goals to their study, for instance attaining their undergraduate diploma in due time, or getting high grades. some had intermediate study delays, but sooner or later commitment often grew and study became a priority.

*Now, my school is very important, I don’t want to do retakes, because I can’t choose another study again. My student grant ends at some time and anyhow I have to pay the next three years myself. I want to do it well and timely, not being 30 when I graduate. Imagine I’m 30 and by then I have to start my career, find a husband and possibly have kids. And that has to happen before a certain age. That’s also why I want to pass my exams in one time*. (Kim, I1, DDD)

In their narratives, interviewees often expressed commitment to study and work, and to strive for a steady job career. Evidently, the more important participants considered their study or job, the more effort they put into it and the more committed they felt.

*When I’m at work, I’m ambitious. In my last job I got promoted to supervisor within one year, and that is something I want to achieve. I have higher aspirations, and I cannot simply work somewhere for 8 hours and watch the clock. I envy people who are able to do that: have a job, do their work and that’s it. I am not like that; my work always follows me home. Yeah, I’m pretty ambitious*. (Kevin, I2, NNN)

### Cannabis use in relation to study and work

Most interviewees believed that heavy cannabis use would negatively impact their daily occupational functioning and most of them had experienced adverse effects themselves, such as difficulties getting out of bed the next day, functioning more slowly and sloppy, trouble memorizing, and postponing tasks. However, almost one in five participants (8/47) reported better functioning in some tasks when being high or stoned, mainly because they believed it improved their concentration. With cannabis, they felt like being “in a bubble” and less distracted by other people, actions or thoughts. Interestingly, all these interviewees stated to have ADHD and/or ADD (all except one clinically diagnosed), and some said that cannabis was like “natural Ritalin” or a kind of “self-medication.”

“*Recently I finished that training, and started my own company. It goes really well. I’m much more concentrated in my work after using cannabis. And when I’m programming when I’m stoned, I’m like in the codes straight away, type everything effortlessly. Sober I start thinking about how it’s working, the syntaxes, commando’s, but stoned all of that happens fully automatic. I get into a kind of vibe to program completely uninterrupted. It makes a big difference*.” (Ben, I1, DNN)

Almost all student and employed interviewees took it for granted not to use cannabis before or at school or work or when studying mainly to avoid adverse effects and/or out of responsibility.

*Interviewer: Why don’t you smoke cannabis at work? Interviewee: Well, it’s kind of … On the one hand I think that they wouldn’t be cool with that. I think they want to hire the sober Jacob. On the other hand: sometimes, when you have smoked a couple of joints you lose a little attention to details. And that is something that’s really important in my job, the details. So, not using cannabis at work out of feelings of responsibility, but perhaps also to distinguish work from leisure. Like: you’re not here to chill but to work*. (Jacob, I2, NNN)

Other reasons for not using cannabis at work or at school were fear that colleagues would notice it and fear of possible consequences, such as being taken less seriously or being fired. Most interviewees said colleagues or fellow students did not know about their cannabis use. They believed that cannabis use was a private matter, and preferred to keep it to themselves. The dominant patterns in the narratives was to be rather firm in stating that it was inappropriate to be intoxicated while at work, at school or when studying. Using cannabis belonged to the leisure domain, and they reported that they only used cannabis after finishing study or work. As a result, most employed participants barely used cannabis at daytime, and more on weekends than on weekdays. With students, there was more variation, as their daily life was less structured around fixed hours throughout the week. They sometimes used cannabis at daytime, and more often during holidays. Among the participants without study and work, the three that were full time parents sometimes used cannabis at daytime when the children were at school, but more often at night when the children were asleep; they used less or not at all during school holidays.

Despite interviewees generally holding strong views on not using cannabis before and during study or work, some did admit it had happened occasionally. While employed participants seemed to be most strict in not using when at work, students sometimes believed that study differs from work, as there is less social control at college (e.g., when not showing up or not paying attention in classes).

*I am very strict: when I have to work or go to school I don’t smoke. Well, school … occasionally, when my class begins late, at 2 PM, a friend drops by and then we’ll have a cup of coffee and smoke a joint, but not heavy. The first class is also very boring, I go stare out the window or distract others*. (Tess, I1, NDN)

*It’s perhaps more practical not to be too stoned during lectures, but hey, occasionally it doesn’t do any harm. Sometimes, when the lecture begins at 5 PM, well, I sometimes smoke a joint at 3 PM and I think: I shouldn’t have to. I’m trying to take the study really serious, but sometimes it doesn’t work out and I think: oh well, I’ll do it tomorrow. No one is bothered by it; it doesn’t affect anyone*. (Eduard, I2, DDD)

### Relation between study and work events and changes in cannabis use

Not surprisingly given their stage of life, most interviewees reported life events related to study or work that had taken place in the course of our study. In total, participants reported 97 events, averaging 2.1 events per interviewee (Table 2). Four participants reported no events.

**Table 2:** **Events related to study or work**.

Trajectory (*n*) > life event experienced (*n*)	Cannabis use	NNN (12)	NDN (7)	NDD (4)	DNN (10)	DND (2)	DDN (5)	DDD (7)	Total (47)
**T0–T2 (TOTAL)**									
Negatively (van der Pol et al., forthcoming)	More	2	–	3	3	2	3	3	16
	Stable	6	5	–	5	–	–	2	18
	Less	2	1	–	2	–	3	–	8
	Total	10	6	3	10	2	6	5	42
Neutral (5)	More	–	–	–	1	–	–	–	1
	Stable	–	1	1	2	–	–	–	4
	Total	–	1	1	3	–	–	–	5
Positively (50)	More	–	1	1	1	1	1	–	5
	Stable	14	6	1	7	3	3	2	36
	Less	1	1	1	1	–	3	2	9
	Total	15	8	3	9	4	7	4	50
**AVERAGE NUMBER EVENTS PER PARTICIPANT**									
Negatively		0.8	0.9	0.8	1.0	1.0	1.2	0.7	0.9
Neutral		–	0.1	0.3	0.2	–	–	–	0.1
Positively		1.3	1.1	0.8	0.9	2.0	1.4	0.6	1.1
Total		2.1	2.1	1.8	2.1	3.0	2.6	1.3	2.1

Most changes and events concerned starting a new study or job, graduating, finishing a study, quitting work or a study prematurely, and stress related to study or work. Slightly more events were evaluated as positive than as negative. Getting high grades, graduation, and starting a new job always had positive meanings to the interviewees, and starting a new study very often as well. Being fired from a job, getting low grades, and stress were always experienced as negative. Only a few events, although reported as important to interviewees, were perceived as neutral (neither positive nor negative, or both positive and negative), all being study-related (e.g., study delay or starting graduate courses). Quitting a study was experienced the most ambiguously, mainly depending on whether or not this happened voluntary. In line with Rönkä et al. (36), we found that interviewees associated positively experienced events more often with personal choice than negatively experienced events. Nevertheless, interviewees reported almost as many negatively experienced events with little or no personal choice as negatively experienced events where personal choice was present. Over one third of the interviewees reported more than one event, mostly both a negatively and a positively experienced event, such as being fired from work (negative) and getting a new job (positive).

Interviewees talked about changes in their cannabis use in terms of more use (i.e., more frequently, more joints per occasion, or larger amounts of cannabis), or less use (i.e., less frequently, less joints per occasion, or smaller amounts), or said their cannabis use had not changed (stable use). Negatively experienced events were most frequently associated with stable use (43%), somewhat less frequently with more use (38%), and least frequently with less use (19%). In contrast, positively experienced events were most frequently associated with stable use (72%) and much less frequently with less use (18%) or more use (10%). In more than half of the events, interviewees said that they had not impacted their cannabis use. This mainly concerned events that interviewees perceived as positive, but also as planned and not really changing their daily life, or as neutral. As Wheaton and Gotlib (22) stated, “contrast” is important for events to become turning points. In our study, many participants who became graduate students after having attained their bachelor’s degree, although they were surely happy with their certificate, did not change their life drastically. Likewise, employed participants who had switched from a job to a similar one, often considered their new job, although they were pleased with it, as little influential on their daily life. Therefore, these changes in study or work did not really influence their cannabis use.

Generally, increases or decreases in cannabis use were transient, and according to the interviewees these changes in cannabis use largely depended on changes in the amount of leisure time that went along with events or temporary changes. For instance, becoming unemployed or having a quiet study period led to more leisure time and thereby more cannabis use, whereas a new job or a busy study period led to less leisure time, and consequently to less cannabis use.

*[about the timeline] The more demanding my study, the lesser I smoke. When I’m free, there is a peak in my use. Let’s see. In June and July I’ve used less, because I worked at a bank for 2 months, nine-to-five job, little leisure time. Then in August, an increase in use, like “long live freedom! Now I can smoke again”. After that, a normal level for a while. December slowly a decrease, because then the exams come closer. January a drop, heavy times and tough exams, 4-5 exams in one week, so then it’s 0-1 joint per day. And then February suddenly again ‘freedom!’, so daily use, 2 joints anyhow*. (Zoë, I1, NNN)

*When I have a lot of leisure time, I smoke more and sooner. When I’m busier and more serious, then I smoke less. And that is certainly a correlation, when there is an ascending line with responsibilities and working hard, there is simultaneously a descending line with cannabis use*. (Robert, I2, DDN)

In addition, agency came to the forefront as an important factor, most clearly in the narratives of students. Several students reported considerable delay in their study, which they all experienced as negative and some were facing a demanding last year of studies. Some expressed a low level of agency regarding their study, did not feel in control, gave up, and subsequently started to use more cannabis.

*I felt really bad that period. I did go out with friends, but I didn’t do much for my study and I only worked now and then. I didn’t give it my all. I smoked a lot and I started to use that, as an excuse. I had no priorities, things just happened. Life happened to me, and I sort of endorsed it*… (Julian, I2, DDD)

In contrast, other students chose and managed to restructure their daily life and to study hard, and, although they did not necessarily blame their study delay on cannabis, they actively reduced their cannabis use. They all stated that they were highly motivated to change their cannabis use and were convinced that they could succeed. In the course of our study, three participants reported to have quit using cannabis, giving their occupational life as the main reason, as they thought cannabis was not conducive to their functioning. They said that quitting did not occur overnight, but was a gradual process: they went from daily use to only in weekends, and step-by-step cut back. At the last interview they had not used cannabis for over a year and neither had the intention to start again.

*My medical study was suffering from my cannabis use. Whenever I have an exam I have to study very hard, a full week every day, spending the whole day in the library, otherwise I won’t make it. When I was using cannabis, being there at 8:30 AM was a problem anyhow, because I couldn’t wake up early. Also, after 3 PM I didn’t feel like studying anymore, no concentration, I wasn’t able to memorize things. Factual knowledge doesn’t go together with cannabis use. I always stopped using a week before the exams, but you need three days to get active and to get adjusted, and in fact you’re too late. Also, smoking cannabis at night does not go well with lectures early in the morning. I often overslept and didn’t go. All in all my study delay was one year. Last year, I decided: I don’t want to use cannabis, I want to catch up on my study. And I did! Now I do great, I pass the exams, so I shouldn’t smoke anymore. The difference between when I was smoking cannabis and now is huge.(…) I feel in control of my life now more than ever*. (Sofie, I1, DDN)

Of the six participants who lost their job during our study, no one reported this was related to their cannabis use. While one could argue that cannabis may have affected their functioning and thus indirectly caused job loss, this did not seem the case as mostly their dismissal was due to cut-backs related to the crisis.

### Relation between stress and changes in cannabis use

A recurring topic in many narratives was stress related to study or work, though not *per se* in conjunction with events. For students such stress mainly involved study delays and exam periods, especially their final project or master thesis. For employed interviewees it was largely connected with deadlines, having to work too many hours and reorganizations or job loss. Participants without study or work perceived stress mainly related to financial problems and sometimes parenting. Stress came with ups and downs, and could have a strong impact on participants’ mood and everyday life functioning, including cannabis use. Some interviewees explained how cannabis use could be functional in dealing with stress, because it helped them to distract their mind, making it easier to relax and taking a moment for oneself. For some, smoking a joint at the end of the day was also a reward for their hard work. Consequently, it was not uncommon for interviewees to explain increases in their cannabis use by stressful and busy times.

*When I’m stressed, or more stressed, then I’m gonna smoke more. Just to forget a bit. It won’t solve anything, but for the moment it does, you can simply let things go*. (Samantha, I2, NNN)

*When I’m stressed, the urge to smoke increases. I don’t know if that’s positive or negative, probably not positive, but hey, it gives me some peace. By then I think: ok, now I have a break, it’s ok now. If I don’t have that break I’m a bit stuck with that frustration. Additionally, it relaxes me. Except that the next day at work I’m a little less alert and probably it’s not beneficial, but at least it relieves the evening itself*. (Jonas, I2, DDD)

Conversely, other participants explained a decrease in their cannabis use by stressful times. Some thought that with stress cannabis use was not helpful, since it might intensify emotions and lead to more stress or worries. For some others, like Kevin, using less cannabis in times of stress was not so much because of possible unpleasant effects, but primarily a matter of time and personal choice.

*Interviewee: At that time I used less. See, when you’ve had a really busy day and you come home at 8 PM and you want to go to the gym and cook a meal and also have to smoke a joint and get up at 7h the next morning, no, that won’t work. Interviewer: To what extent is it about priorities? Interviewee: Yeah, it depends on your priorities, but for me it’s not cannabis, I prioritize my job. No, when I’m stressed I’m not going to smoke more, but less instead*. (Kevin, I2, NNN)

In five participants, chronic stress ended in a situation of “burnout.” They all experienced this as very negative and it took them at least a couple of months to recover. Two of these interviewees thought their cannabis use was worsening their mental health and stopped using (one permanently and one temporarily with the intention to quit permanently). One of these interviewees remained stable in her cannabis use and two others used more cannabis during their burnout and said that this was because they had more leisure time.

### Relation between study and work events and cannabis dependence trajectories

Regarding cannabis dependence, seven different trajectories evolved, with persistent non-dependent (NNN; *n* = 12) and transitions from dependent at baseline to non-dependent at T1 and T2 (DNN; *n* = 10) being the most common trajectories (Table 1). On average 2.1 events were reported, but this was only 1.3 in the group of persistent dependent participants (DDD; *n* = 7; Table 2).

Although numbers of participants in most trajectories are small (*n* = 2–12), some patterns seem to become manifest. In response to occupational events, interviewees who were non-dependent at T2 (NNN, NDN, DNN, DDN) mostly had not changed their use (49/75 events) or rather equally often used less (14/75) or more cannabis (12/75). Interviewees who were dependent at T2 (DDD, DND, and NDD), though they also quite often said that their cannabis use had not changed because of events (9/22 events), were somewhat more likely to use more (10/22) than less (3/22).

Concerning occupational status (study, work, or neither) and trajectories some interesting patterns emerged. Firstly, many participants remained student during our study (23/47) and, although they can be found in six different trajectories, the overall tendency over time is away from cannabis dependence (Table 3). Four of these students were persistent non-dependent (NNN). While 14/23 participants who remained student were dependent at T0, only five were at T2. In general, the students who became non-dependent (7 DNN, 4 DDN, 3 NDN) stated that their study became more demanding as it progressed, which they found difficult to combine with frequent cannabis use. From their narratives it became clear that they decided for more control over their cannabis use, through being more selective in when to use and when not and/or through less frequent use.

*I concluded for myself that if I really want to succeed in life, I have to fully go for this study now. And that has changed my cannabis use as well. I still use, every week I do, but not daily anymore. Because when I do, the next day I don’t feel alert, I notice I can’t really concentrate. That interferes with what I want to do, my study. So now I only smoke in the weekends, or when I don’t have any obligations the next days. I plan my use, take it into account. More seriously. My study is the first priority now, definitely. From February till June 2011 it wasn’t, and I used cannabis very often. That was less serious, I wasn’t devoted to my study and I attended the university mainly to socialize*. (Max, I2, DDN)

**Table 3:** **Occupational status and cannabis dependence trajectories T0–T1–T2 (*n* = 47)**.

T0	T1	T2	NNN (12)	NDN (7)	NDD (4)	DNN (10)	DND (2)	DDN (5)	DDD (7)	Total (47)
Study	Study	Study	4	3	2	7	–	4	3	23
Study	Study	Work	1	1	–	–	–	–	–	2
Study	Work	Work	3	–	–	2	–	–	–	5
Work	Study	Work	–	–	–	1	–	–	–	1
Work	Work	Work	2	2	2	–	1	1	2	10
Neither	Study	Study	1	–	–	–	–	–	–	1
Neither	Neither	Neither	1	1	–	–	–	–	2	4
Study	Work	Neither	–	–	–	–	1	–	–	1

In contrast, four of the five participants who remained student and who were dependent at T2 (3 DDD, 2 NDD) expressed in their narratives a lower level of agency regarding their study, e.g., reported that they did not take their study very seriously, or did not spend enough time on it.

*I can’t convince myself of the need to quit using cannabis. I don’t encounter adverse effects. There are things, such as my study delay, that cannabis contributed to. But the real decisive factor is if I really had the willpower and would go for it, then I would succeed in my study. Even when using that much cannabis. It’s just my own laxity I think. I have had that my whole life*. (Eduard, I2, DDD)

Secondly, all seven students who became employed, either after quitting their study (by choice or involuntary due to poor performance) or after graduation, were non-dependent at T2. Four (with stable or reduced cannabis use) showed a persistent non-dependent trajectory (NNN) and three shifted from dependent to non-dependent (1 NDN, 2 DNN) in the same period as their occupational status changed from student to employed. Although this shift co-occurred with change in occupational status, it was not necessarily induced by events related to study or work. Mike (DNN), for example, said that between T0 and T1 he felt that the use of cannabis sometimes made him a bit paranoid. Therefore he decided to decrease his cannabis use, and finally he quit. In the meantime he discontinued his study and started working fulltime. Similarly, Isabel (DNN) expressed that the way she used cannabis evolved as part of a general change in lifestyle rather than specifically because of a shift in occupational status from study to work.

*Like with other things, you need to find a certain balance in cannabis use. For cannabis I have found that balance, I guess. I have that for a year now. Also because I live on my own now, I really got to know myself. You’re alone, there is nobody else around. It has changed me, made me more independent*. (Isabel, I1, DNN)

Regarding the group that remained employed (10/47), no clear patterns in trajectories could be observed. These participants were represented in six different trajectories (2 NNN, 2 NDN, 2 NDD, 1 DND, 1 DDN, 2 DDD). At T0 this group included four dependent participants versus five at T2. The extent to which employed participants said that they were committed to their job varied, and also their type of job, but this did not appear to be related to their cannabis dependence status. However, sometimes change in cannabis use did not result in a dynamic trajectory, as was the case with Jonas, who stated that over time he had taken more control over his cannabis use, but was diagnosed as persistent dependent (DDD).

*The regularity got out of my cannabis use. I used to smoke every day, a joint before bedtime, perhaps one in the early evening and when I had a day off I could sometimes start in the afternoon. Well, that’s not really something to be proud of, and I always thought: if I want, I can stop using. It was time to prove that. It was a rude awaking [laughs]. Before, I didn’t try to control my use, I never saw the need to. But I began to feel the effects: the relatively easy college life was over, employed life was more demanding, and I had to better take care of myself. Perhaps I still don’t fully regulate my use, I sometimes have relapses. It’s difficult, because after I haven’t been smoking for a while, I think: why not smoke? I don’t have any problems with my use, I’m functioning fine, also when I smoke. I can do my job well, or quite well and my social life as well*. (Jonas, I2, DDD)

Also in the case of the other participants (7/47) no consistent patterns could be observed in the relationship between cannabis dependence trajectories and (events in) the occupational domain. Alternatively, agency, more specifically their ability to regulate their cannabis use appears to be related to (transitions in) their dependence status. This became most clear for three participants with young children in the neither group (NNN, NDN, DDD). During our study these three mothers experienced the event of one or two children going to school for the first time, which created a considerable change in their daily time schedule. Although they all underlined not to use cannabis in presence of their children, the way they organized their cannabis use was quite different. Samantha (NNN) believed to be in control over her cannabis use. She used cannabis mainly at night, before going to sleep, and only after she had taken care of her daily responsibilities. Contrariwise, Charlotte (DDD) said that her kids often arrived too late at school, because she had difficulties getting up in the morning, and that she smoked a joint right after she had brought her children to school, even though she knew that by doing so she often postponed her daily tasks. She felt addicted, not in control over her use and in both in-depth interviews she said she would want to quit. Nathalie (NDN), on the other hand, often used cannabis after having finished her daily tasks, but between T0 and T1, when her son started to attend school, she experienced a period that she used more frequently and also in the morning. In retrospect, she believed during that time she was addicted to cannabis, and she had decided to change her use and to (successfully) retake control over it.

## Discussion

In this qualitative study we explored the role of study and work in cannabis use among a group of young adult initially frequent cannabis users. We were particularly interested in analyzing how study and work, and more specifically events related to these domains, contributed to transitions in cannabis use and dependence. We interviewed 47 young adults in-depth twice retrospectively covering a period of 3 years. All interviewees were frequent cannabis users at the start of the study (T0). During the follow-up period, there were wide variations and strong dynamics in their patterns of cannabis use, the presence of cannabis dependence, and their occupational situation. Overall, there was a declining tendency in frequency and quantity of cannabis use, including a few interviewees who had quit using cannabis altogether at the second in-depth interview. Various trajectories concerning cannabis dependence appeared. One quarter of the sample remained persistent non-dependent during the study. Some participants were persistent dependent, and others switched from a dependent to non-dependent status and vice versa, yet, at the end of the study more participants were non-dependent than at baseline (34 versus 23 of all 47 interviewees).

Almost two-thirds of the interviewees were students (often with a job on the side) at baseline and remained student during the total study period. Most other participants were in paid employment, and in the course of our study some students became employed as well, indicating that long-term frequent cannabis use does not necessarily restrain individuals in their professional life [cf. (37, 38)]. Most interviewees considered cannabis use as inappropriate before or during hours of study or work [cf. (39)].

As expected in this age group (mean age 21 years), life events related to study or work were quite common, nearly all participants experienced at least one such an event. Overall, participants evaluated slightly more events as positive than negative. Similar events could be valued differently, and it was evident that agency did matter. In line with Rönkä et al. (36), events were likely to be experienced positively when personal choice was felt to be present, e.g., when students decided themselves to discontinue a study rather than being forced to stop, or when individuals choose to start a new job rather than being fired. Our study shows that events in the context of study or work have the potential to, but not necessarily do, influence cannabis use. It should be noted that events that did have an impact on cannabis use often were gradual rather than abrupt, and often cannabis use changed gradually. The feeling of being in control, i.e., agency, in the case of occupational events also appeared relevant for cannabis use. Many events did not lead to changes in cannabis use, but negatively experienced events were mainly associated with stable (43%) or more (38%) cannabis use, whereas positively experienced events were mainly associated stable (72%) or less (18%) cannabis use. Our findings further suggested that increases or decreases in cannabis use related to occupational events are at least partly explained by changes in the amount of leisure time. For example, participants tended to report more use after becoming unemployed, while those who started a new job reported less cannabis use. Changes in cannabis use were also explained by job and study-related stress and how interviewees managed stress. Some reported less use, because using while stressed would enhance negative emotions, or simply because of too little time left to use. Conversely, others reported more use in stressful periods, because cannabis helped them to relax, or was a reward at the end of a day of study or work.

We also found indications for reverse causation, i.e., changes in cannabis use can lead to changes in study or work. Several interviewees, because of events such as study delays, or (expected) stressful times, gradually managed to rigorously cut back or even quit their cannabis use, which eventually was conducive to their occupational performance. Overall, interviewees, who considered their study or work as being rather important, were more committed and motivated and were more willing to rule out any possible influence of their cannabis use on their occupational functioning.

Inspections on occupational events in relation to cannabis dependence (trajectories) revealed that in response to events, participants who were non-dependent at T2 mostly had not changed their use, or equally often used less or more cannabis. In contrast, interviewees who were dependent at T2 were more likely to use more rather than less in response to (negative) occupational events. Besides, interesting patterns emerged concerning occupational status (study, work, or neither). Among participants who remained student during our study, the overall tendency over time was away from cannabis dependence. The students who switched to non-dependence found their study, as it progressed and became more demanding, hard to combine with frequent cannabis use and decided for more control, through being more selective in timing and frequency of use. All students who became employed during our study were non-dependent at T2. Besides, none of the students who entered the workforce were dependent at T2, although the transition was not necessarily induced by study or work events.

For other participants, including those who remained employed, no clear patterns in trajectories could be observed. Alternatively, agency, more specifically their ability to regulate their cannabis use, appeared to be related to (transitions in) their dependence status.

Taken together, our study supports a reciprocal relationship between occupational life (events) and frequent cannabis use and dependence. On the one hand cannabis use and dependence impact occupational life either negatively, in terms of worsened occupational functioning, or positively, e.g., when users deliberately cut back on or stop using cannabis to improve their professional performance. On the other hand our findings support Laub and Sampson’s (23) line of reasoning that employment and education impact cannabis use and (indirectly) dependence by limiting leisure time and facilitating structure resulting in attenuated cannabis use. However, it could be argued, and as indicated by our findings, that the available leisure time is influenced by several factors, such as the way participants give meaning to their life and study or job, including motivation, priorities, and agency. For example, some interviewees prioritized study over cannabis use and thereby had less leisure time, while others prioritized cannabis use over study, thus had more leisure time. This might require a certain level of agency, i.e., feelings of being in control or believing in one’s own capabilities. In this perspective, the restricting impact of leisure time on cannabis use might be ascribed to the amount of leisure time one *has* as well as to the amount of leisure time one *creates* to use cannabis. As our findings show the relationship works both ways, this provides a nuance for the debate on the “amotivational syndrome.” Our study also supports previous research stating that occupational stress can bring about an increase in drug use (16), yet, might depend on the person (characteristics) experiencing it. For some participants cannabis use was a way of managing everyday demands [see also (37, 38)] or coping with psychiatric symptoms. Especially for AD(H)D participants, cannabis use may reduce symptoms, attenuate sleep problems, and improve social functioning (self-medication) (40). Regarding the relationship between stress, depression, and cannabis use, this self-medication hypothesis – and its potential contra productive effect – is somewhat supported by our quantitative findings that coping motives (although not specifically for depression) were one of the few cannabis related differences between dependent and non-dependent frequent users (8), a predictor of cannabis dependence onset (41), and a predictor of dependence persistence (van der Pol et al., forthcoming).

### Limitations

Our findings add to the growing insight into the relationship between occupational life and cannabis use of young adult cannabis users. Nonetheless several factors might limit the results of this study.

An enriched sample was selected, and therefore we cannot guarantee representativeness. However, this does not necessarily mean that the sample is highly biased. Our sample includes many students, but being a student is rather common for young adults in the Netherlands. Cannabis use and occupational status in our study were quite dynamic, but to some extent this was affected by the study design. We deliberately included dynamic dependence trajectories between T0 and T1 for in-depth interviews. More generally, our sample of young adults is likely to be dynamic or even volatile in different aspects, including education and employment. From the life course theory perspective, a decline in cannabis use during young adulthood was to be expected with aging.

Moreover, we investigated the process of cannabis use in the periods between interviews (T0–T1–T2), whereas cannabis dependence was dichotomously captured in diagnoses of dependence versus non-dependence, based on the presence of symptoms within a certain period. Not only could much variation underlie these diagnoses, since they refer to the time between two interviews, also the “effect” of an event related to study or work on cannabis dependence might not have been revealed, and only become apparent afterward, in a next interview. Likewise, participants who had stopped using were categorized as non-dependent, while they were actually non-users.

Furthermore, it should be noted that some results presented here may not be universally replicable because they are related to the country where the study is conducted. Dutch policy officially tolerates possession and sale of small amounts of cannabis, and this may limit extrapolation of our results to countries with formal penalties. Yet, we intended to explore in-depth the role of study and work in cannabis use and dependence rather than to portray a representation of all cannabis users. Although research suggests cannabis laws have little impact on cannabis use patterns of regular users [e.g., Ref. (42–44)], their experiences of certain life events, feelings of personal choice and control, and therefore the outcomes of life events might be indirectly affected by cannabis policy. Hence, a comparable study in another country might therefore find different results.

Finally, as mentioned before, our analyses are based on the narratives of the interviewees, and they largely create their own reconstructions of their cannabis careers and lives. Consequently, their self-perception and self-reflection formed the foundation of our analyses and interpretations. It should be noted that when interpreting the results, all data were based on self-report. We mainly looked into the subjective, not objectified, meanings of (occupational) events. Although subjective, participants’ evaluation of events often corresponded with how one would categorize them objectively (from an outsider’s perspective). Also the use of context-based timelines, including data participants (quantitatively) reported intermediately, positively contributed to the recall of their lives and cannabis use. More importantly, our approach gave novel insights in the perceptions, experiences, and attributed meanings of participants, which is reflected in the emerging importance of agency in the narratives. For example, although many interviewees stated that they had to learn by their own experience how cannabis use can impact job or study performance, most prioritized their obligations, out of personal motivations or an overall strong work ethic.

How can we explain that occupational events left cannabis use largely unchanged? An explanation could be that for young adults, events such as a new study or a job switch are quite normal and part of a normal career. In fact, sometimes these events were not changing participants’ daily lives. Besides, cannabis use appeared to be primarily a leisure activity. These findings relate to the normalization thesis, which suggests that in the past decades, for many users cannabis use has become a normal part of their life, which includes clear choices about whether, where and when (not) to use (45, 46). Cannabis use assimilates quite well with studying and/or being employed, but rules and norms are applied: users do not use cannabis just anytime and anywhere. Cannabis is preferably not used with colleagues and is reserved for leisure time. In this study we focused on the professional life domain, thereby somewhat artificially taking this domain out of its wider context. Life events in other domains, for example social relationships with relatives, partners, and friends, might be equally or even more important.

Life course theory appeared a useful framework to explore how and why education and employment are related to cannabis use and dependence over time. Our study showed that life events in the realm of education and employment were rather common in young adults’ lives and can have a strong impact on their cannabis use. Changes in cannabis use are sometimes temporary, but turning points in cannabis use careers can evolve from events in education and employment, as became most clear for the interviewees who fully quit using cannabis. To conclude, and similar to desistance from crime, cessation of cannabis use often is a gradual process, in which agency plays a major role. Besides, regarding the occupational life of young adult cannabis users, leisure time is a (important) factor underlying changes in frequent cannabis use.

## Conflict of Interest Statement

The authors declare that the research was conducted in the absence of any commercial or financial relationships that could be construed as a potential conflict of interest.
